# From fibrotic mechanisms to clinical translation: the drug therapy revolution and delivery system breakthrough in proliferative vitreoretinopathy

**DOI:** 10.3389/fimmu.2026.1774802

**Published:** 2026-02-10

**Authors:** Zijian Chen, Jiangying Liu, Liao Quan, Lingdan Wu, Qihua Xu

**Affiliations:** 1 School of Optometry, Jiangxi Medical College, Nanchang University, Nanchang, Jiangxi, China; 2 Jiangxi Research Institute of Ophthalmology and Visual Science, Nanchang, Jiangxi, China; 3 Jiangxi Provincial Key Laboratory for Ophthalmology, Nanchang, Jiangxi, China; 4 National Clinical Research Center for Ocular Diseases Jiangxi Province Division, Nanchang, Jiangxi, China; 5 The Affiliated Eye Hospital, Jiangxi Medical College, Nanchang University, Nanchang, Jiangxi, China; 6 Jiangxi Clinical Research Center for Ophthalmic Disease, Nanchang, Jiangxi, China

**Keywords:** proliferative vitreoretinopathy, EMT, mechanism, cytokine, therapy, drug delivery system

## Abstract

Proliferative vitreoretinopathy (PVR) is a severe fibrotic complication following ocular trauma or retinal detachment surgery, characterized by complex multicellular and multicytokine interactions. Its core mechanism involves retinal pigment epithelial (RPE) cells, glial cells, and inflammatory cells undergoing epithelial-mesenchymal transition (EMT), abnormal proliferation, migration, and contraction, ultimately leading to traction retinal detachment. Current treatment primarily relies on surgery, but faces limitations such as high postoperative recurrence rates and a lack of clinically approved effective adjuvant drugs. This review provides a comprehensive analysis of the PVR pathogenic network, integrating the roles of diverse cellular players with key signaling pathways. We place a particular emphasis on critically evaluating emerging therapeutic strategies, including targeted pathway inhibitors and, notably, innovative drug delivery systems, which represent a paradigm shift towards overcoming pharmacological barriers. By synthesizing mechanistic insights with translational applications, this article highlights current advances and underscores the gap between preclinical promise and clinical efficacy. It aims to serve as a timely resource for understanding the pathobiology of PVR and for guiding the development of future multi-targeted therapeutic interventions.

## 1 Introduction

Proliferative vitreoretinopathy (PVR) is a blinding eye disease characterized by abnormal scar formation. Following retinal detachment or ocular trauma, damaged retinal pigment epithelial (RPE) cells migrate into the vitreous and interact with glial cells, vitreous cells, macrophages, and fibroblasts to form fibrous membranes (1). This process is termed epithelial-mesenchymal transition (EMT) (2). As extensive fibrous membranes proliferate and contract, they ultimately cause traction retinal detachment (3). Surgical removal of PVR membranes remains the sole treatment option, yet surgical outcomes are poor with a high recurrence risk (4). Studies indicate that the surgical success rate for retinal detachment (RD) complicated by PVR is only 69-75% (compared to 98% for uncomplicated RD), and patients with PVR face poorer visual prognosis (5). Furthermore, PVR patients often undergo multiple ocular surgeries, significantly increasing the financial and psychological burden on patients (6).

EMT is the process by which epithelial cells lose their epithelial properties and transform into mesenchymal cells upon specific stimulation, representing a critical step in the development and progression of PVR (2, 7). Multiple signaling pathways are involved in EMT, including transforming growth factor-β (TGF-β), NOTCH, Wnt/β-catenin, and receptor tyrosine kinases (8–11). As research into PVR deepens, an increasing number of pathways and molecules are being identified, offering new opportunities for the development of therapeutic drugs targeting PVR. To date, numerous drugs have demonstrated promising results in animal or *in vitro* PVR studies (12–15). However, no therapeutic agent has successfully progressed to clinical application. This failure primarily stems from the complex and poorly defined pathogenesis of PVR, coupled with the dynamic and continuous nature of EMT as a biological cellular transformation process (16). Currently tested drugs fail to precisely target specific stages in PVR progression, resulting in limited efficacy and potential adverse effects for patients (5). For instance, intravitreal injections of dexamethasone or 5-FU are rapidly cleared and may induce retinal toxicity (17, 18). Consequently, targeted therapeutic strategies for PVR have gained critical importance.

Over the years, extensive research has focused on developing novel therapeutic agents for PVR that precisely inhibit the molecular pathways or EMT processes driving its progression. This aims to enhance drug bioavailability at target sites while minimizing adverse effects. This paper delves into the pathogenesis of PVR and reviews current advances in pharmacological treatments. It seeks to evaluate the clinical translational potential and challenges of existing and emerging therapies for proliferative vitreoretinopathy.

The unique value of this review lies in its systematic integration of the roles of various cellular players in PVR—including retinal pigment epithelial (RPE) cells, Müller glia, macrophages, and fibroblasts—with the complex cytokine networks that drive fibrosis. It provides a focused critique of recent advances in targeted pathway inhibitors and offers a comprehensive analysis of innovative drug delivery systems that hold promise for overcoming the pharmacological barriers historically associated with PVR treatment. By closely linking mechanistic insights to translational applications, this review serves as a timely reference for both basic research and clinical practice.

## 2 Blood-retinal barrier disruption and cell migration

Breach of the blood-retinal barrier (BRB) is the primary factor leading to retinal fibrosis and fibrous membrane formation in PVR. In PVR, BRB disruption is primarily triggered by physical injuries such as retinal tears or breaks (19). When the retina is damaged, local inflammation and hypoxic conditions activate inflammatory cells (primarily macrophages, lymphocytes, and polymorphonuclear cells), which trigger PVR development by producing cytokines and growth factors (20). Furthermore, the hypoxic state further compromises the integrity of the blood-retinal barrier. Prolonged separation of the retina from the retinal pigment epithelium (RPE) layer compromises the integrity of the blood-retinal barrier, leading to loss of connections between RPE cells. Subsequently, RPE cells and glial cells (such as Müller cells) migrate from the retina into the vitreous (1). Vitreous fluid contains abundant cytokines and growth factors that stimulate the activation and proliferation of RPE and glial cells (21).

EMT of RPE cells represents a key pathological change in the development and progression of PVR (22). RPE cells form a single-layered epithelial layer between the neuroretina and choroid, supporting retinal homeostasis by participating in the visual cycle, secreting neurotrophic factors, scavenging reactive oxygen species (ROS), maintaining the blood-retinal barrier, and clearing retinal waste (23). Under physiological conditions, RPE cells are non-proliferative (24). However, disruption of the BRB exposes RPE cells to a profusion of cytokines and growth factors within the vitreous. Stimulated RPE cells proliferate, undergo EMT, and acquire the ability to migrate into the vitreous or retinal inner layers through retinal tears. During this process, an extracellular matrix (ECM) containing collagen and fibronectin is produced, and RPE cells progressively transform into myofibroblast-like cells, leading to fibrotic membrane formation (25).

Müller cells proliferate as part of the inflammatory response, aiming to repair the retina to protect the neurosensory retina from mechanical stimulation and safeguard photoreceptors from apoptosis (19). Generally, Müller cells enhance the barrier function of the vascular endothelium by secreting appropriate levels of factors such as pigment epithelium-derived factor(PEDF), thrombospondin-1(TSP-1), neurturin, and glial cell-derived neurotrophic factor(GDNF). Under conditions of hypoxia, mechanical stress, or inflammation, multiple signaling pathways are activated in Müller cells, leading to increased expression of proangiogenic factors such as vascular endothelial growth factor (VEGF), basic fibroblast growth factor (bFGF), matrix metalloproteinases(MMPs), netrin-4, and angiopoietin-4 (26), thereby promoting the development of PVR. Research indicates that Müller cells, like RPE cells, participate in the EMT process. When the retina undergoes mechanical stimulation or detachment, Müller cells migrate to the retinal surface and contribute to the formation of the proliferative membranes (27). Notably, Müller cells serve not only as primary mechanical sensors of the retina but also as major producers of ECM components (28). Studies reveal that PDGF is massively released from retinal injury sites, inducing activation of Müller cells and other glial cells. This exacerbates ECM production and retinal adhesion while enhancing the mechanical strength of proliferative membranes (29).

Additionally, following disruption of the BRB, macrophages enter the vitreous cavity through retinal lesions and release inflammatory cytokines, stimulating cell migration and proliferation (30). At this stage, M2 macrophages are generated either through the differentiation of recruited infiltrating monocytes or the polarization of infiltrating M1 macrophages (31). M1 macrophages exhibit a pro-inflammatory phenotype, secreting pro-inflammatory cytokines including IL-1β, IL-6, IL-12, IL-23, and TNF-α (32). STAT6 is activated during this period, promoting IL-4/IL-13-mediated M2 macrophage differentiation by upregulating Arg-1 and various other pro-fibrotic phenotypic genes (33). M2 macrophages exhibit an anti-inflammatory phenotype but can stimulate fibroblasts to increase ECM production. The degradation of ECM releases matrix-associated growth factors such as TGF-β and cytokines, promoting fibrosis (32). Furthermore, *in vitro* analyses demonstrate that microvesicles secreted by M2 macrophages promote proliferation and migration of RPE cells by activating the PI3K/AKT/mTOR signaling pathway.

Fibroblasts are circulating bone marrow-derived progenitor cells first identified in experimental wound healing models (34),and subsequently demonstrated to play a critical role in both normal and abnormal human healing (35). Fibroblasts express CD34, CD45, collagen I, and vimentin. However, upon exposure to TGF-β or endothelin-1, fibroblasts differentiate into myofibroblast-like cells, expressing Alpha-Smooth Muscle Actin(α-SMA), producing fibronectin and collagen, and losing CD34 and CD45 expression (36–38). Myofibroblasts possess contractile properties, and the products they express directly promote the onset and progression of fibrosis in PVR (39).

Myofibroblasts in PVR primarily originate from (1): direct activation of intrinsic stromal fibroblasts within the eye (40) (2);transformation of RPE or Müller cells during EMT (41) (3); Circulating fibroblasts entering the PVR retina function as progenitor cells for myofibroblasts (42). Notably, recent studies have revealed that myofibroblasts in PVR can form via macrophage-to-myofibroblast transformation (MMT), a process involving predominantly M2 macrophages. Ahmed M et al. demonstrated that CD206+ M2 macrophages express myofibroblast-consistent markers including α-SMA, fibroblast-specific protein-1 (FSP-1), fibroblast activation protein-alpha (FAP-α), and β-catenin (39). Furthermore, contractile myofibroblasts within PVR membranes may also originate from undifferentiated Myo/Nog cells (43, 44). The retina normally contains a small number of Myo/Nog cells, and hypoxia and light injury stimulate their proliferation and migration toward areas of stress and cell death (45). Gerhart et al. demonstrated the presence of Myo/Nog cells within the fibrous membranes of PVR patients, expressing α-SMA and striated muscle myosin alongside low levels of myogenic differentiation (MyoD) (44).

**Figure 1:**
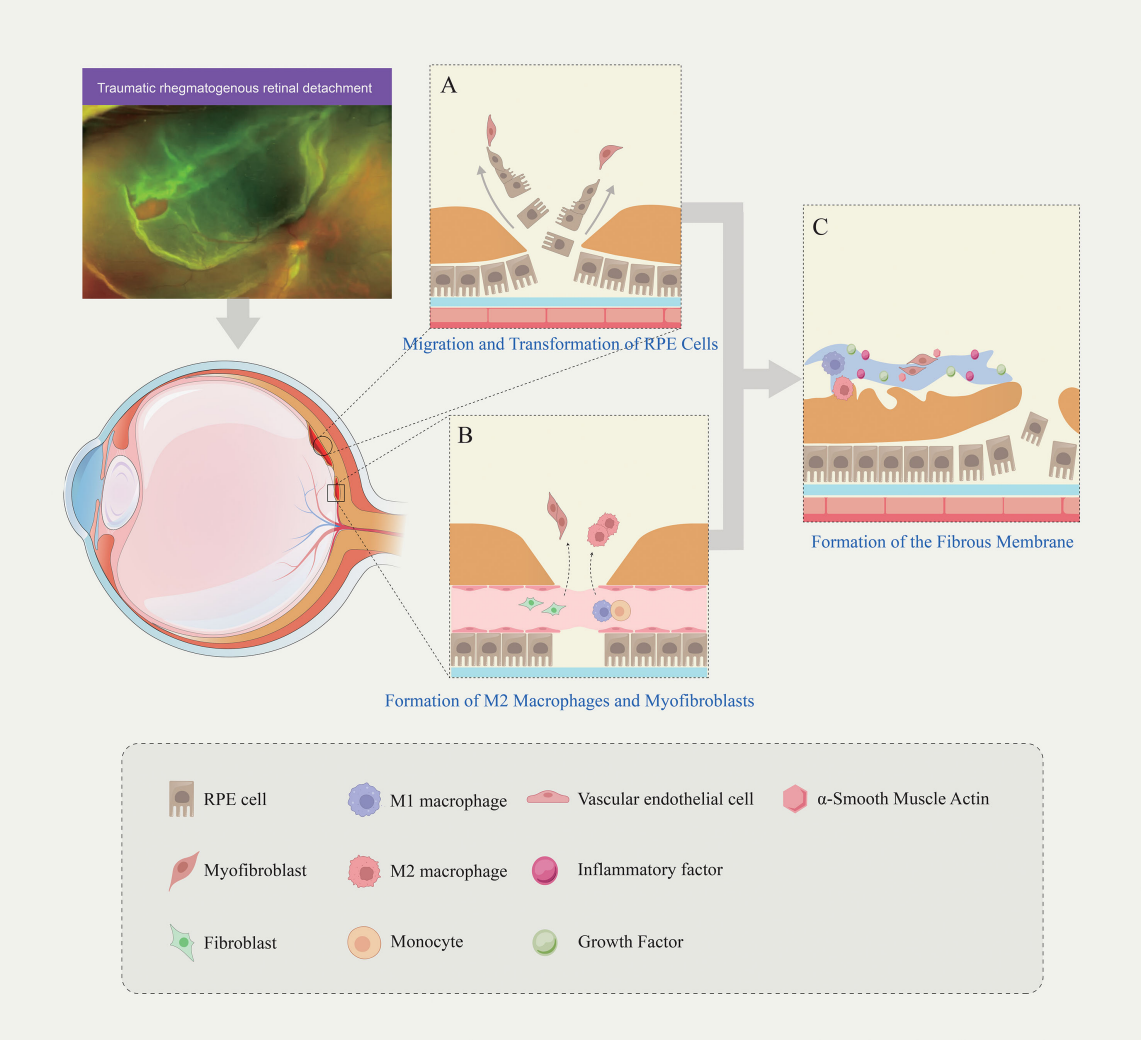
Cell migration, transdifferentiation, and fibrous membrane formation during PVR. **(A)** Following ocular trauma or retinal detachment, RPE cells lose their adhesive phenotype, migrate toward the vitreous, and transdifferentiate into myofibroblasts; **(B)** Circulating fibrocytes differentiate into fibroblasts entering the vitreous; M2 macrophages arise either through differentiation of recruited infiltrating monocytes or polarization of infiltrating M1 macrophages; **(C)** Fibrous membranes are collectively formed by multiple cell types (RPE cells, glial cells, myofibroblasts, inflammatory cells), associated cytokines, and substantial extracellular matrix (collagen, fibronectin, etc.).

The complex interplay of cellular migration and transdifferentiation events described above is schematically summarized in **Figure 1**, which provides a visual overview of the key cell types and their roles in fibrous membrane formation during PVR.

## 3 Initiation of fibrosis and fibrotic products

During EMT, RPE cells transition from their normal cuboidal morphology to the characteristic spindle shape of fibroblasts. This transformation involves loss of tight junction polarity, with marked downregulation of zonula occludens-1 (ZO-1) and E-cadherin expression (46, 47). Conversely, the upregulation of α-SMA, fibronectin (FN), and N-cadherin suggests the transformation of RPE cells into myofibroblasts (47, 48). Analysis of human PVR membranes indicates that most α-SMA-expressing myofibroblasts originate from keratin-positive RPE cells (41), which confer contractile properties to the fibroblast membrane. Furthermore, studies have documented the presence of FN in the retinas and vitreous of PVR patients (49, 50).

As mentioned earlier, myofibroblasts are contractile fibroblasts converted during the EMT process, playing a crucial role in wound closure and tissue contraction. The generation of contractile force is primarily associated with α-SMA expression (51). α-SMA is transiently expressed in cardiac and skeletal muscle during embryonic development and is highly restricted to smooth muscle cells (SMCs) in adult animals (52). However, it is also expressed in myofibroblasts during wound healing and in a limited number of adult tissues (53). Studies indicate that TGFβ and contact injury exert synergistic effects at the α-SMA promoter level (54).

As a three-dimensional biological network, the ECM provides cells with a microenvironment to maintain homeostasis, growth, tissue formation, and repair (55). The ECM comprises collagen, fibronectin (FN), laminin, elastin, proteoglycans (PGs), glycosaminoglycans (GAGs), and several other glycoproteins (56). The pathogenesis of PVR involves ECM remodeling and abnormal deposition (57). Among these, collagen I is the predominant collagen fiber in PVR specimens, typically physically associated with RPE and Müller cells (41). Furthermore, MMPs have been detected in the subretinal fluid and vitreous of patients with PVR complicated by retinal detachment. MMPs participate in various cellular and extracellular events, such as ECM degradation and remodeling, wound healing processes, and neovascularization (58). MMP activity is negatively regulated by endogenous tissue metalloproteinase inhibitors (TIMPs) (59). The MMP/TIMP balance is critical for maintaining ECM homeostasis. Notably, both MMP-3 and TIMP-1 have been reported in the retina during PVR (60).

Cadherins play a pivotal role in PVR, primarily participating in processes such as cell-cell adhesion, migration, and EMT, making them crucial molecules in the development and progression of PVR (47). Under normal conditions, epithelial cells (such as RPE cells) predominantly express E-cadherin to maintain the epithelial phenotype and stable cell junctions (61). During EMT, E-cadherin expression is downregulated, while expression of N-cadherin and others is upregulated. This switch alters intercellular adhesion properties: shifting from stable, strong epithelial adhesion to more dynamic, migratory mesenchymal cell adhesion (62, 63). This facilitates RPE cell detachment from the epithelial layer, migration, and transformation into contractile myofibroblasts. Research indicates that loss of E-cadherin leads to the dissociation of β-catenin from the β-catenin-cadherin complex. Subsequently, β-catenin enters the nucleus to activate the ZEB1 gene, thereby promoting the upregulation of N-cadherin (47). Studies also confirm that TGF-β-activated Smad3/4, p38, MAPK, JNK, and ERK1 act as inducers of N-cadherin upregulation (2, 64). Additionally, Ma et al. discovered that CD146 modulates the conversion from E-cadherin to N-cadherin during EMT initiation (65). 

## 4 The role of cytokines in PVR

### 4.1 Expression of growth factors and their related pathways

**Figure 2:**
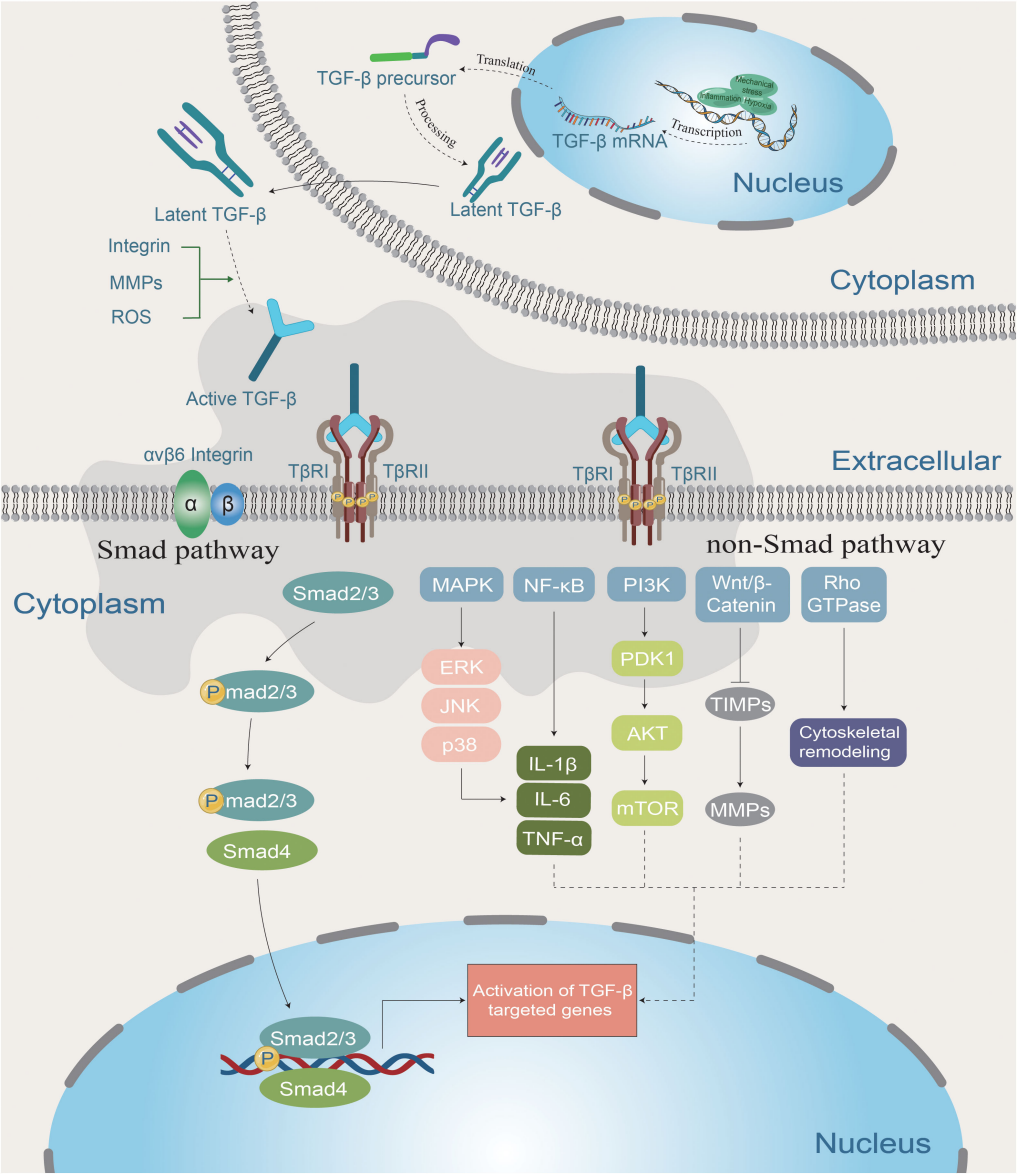
TGF-β induces the development of PVR through classical and non-classical pathways. Upon encountering inflammation, oxidative stress, or mechanical stress, relevant cells induce TGF-β production (inactivated), which becomes activated after stimulation by integrins, MMPs, ROS, etc., participating in both classical and non-classical pathways. The canonical (SMAD-dependent) pathway involves SMAD2/3 phosphorylation and nuclear translocation, while the non-canonical (SMAD-independent) pathway engages signaling cascades such as MAPK, PI3K/AKT, and Rho/ROCK, further driving inflammation and fibrosis.

TGF-β exhibits elevated levels in PVR patients and is considered the primary growth factor inducing RPE cell EMT. Its mediated TGF-β signaling pathway regulates cell differentiation, proliferation, and immunomodulation (66). The pathogenesis of PVR involves chronic inflammation and abnormal activation of the TGF-β signaling pathway, making it a key therapeutic target (67). TGF-β comprises three major subtypes: TGF-β1, TGF-β2, and TGF-β3. TGF-β1 and TGF-β2 primarily contribute to fibrosis and immunosuppression, while TGF-β3 exhibits anti-fibrotic properties under specific conditions (68). Notably, abnormal activation of the TGF-β pathway in RPE cells promotes EMT (69). This pathological transformation is mediated through both classical (SMAD-dependent) and non-classical (SMAD-independent) pathways. The former involves SMAD2/3 phosphorylation and nuclear translocation, while the latter engages signaling cascades such as MAPK, PI3K/AKT, and Rho/ROCK, further driving inflammation and fibrosis (70, 71). Additionally, TGF-β enhances ECM production while inhibiting its degradation (72). The dual signaling mechanisms of TGF-β, encompassing both SMAD-dependent and SMAD-independent pathways, are illustrated in **Figure 2**. This diagram delineates how these pathways converge to drive the inflammatory and fibrotic responses central to PVR pathogenesis.

Connective tissue growth factor (CTGF) plays a pivotal role in PVR (22). CTGF expression in PVR is induced by retinal injury, TGF-β stimulation, and mechanical stress, leading to RPE cell EMT induction, fibroblast proliferation, and excessive ECM deposition (73, 74). Studies reveal that CTGF primarily promotes RPE cell EMT through the PI3K/AKT pathway. With concentration-dependent increases in CTGF, expression levels of epithelial markers (e.g., ZO-1 and E-cadherin) decrease, while expression of mesenchymal markers (including fibronectin, N-cadherin, and α-SMA) increases. This demonstrates the importance of CTGF in the EMT transformation of RPE cells (73).

PDGF functions as a trophic factor during retinal development (75). PDGF is primarily secreted by RPE cells, macrophages, and fibroblasts following retinal detachment or trauma (76). PDGFR (PDGF receptor) has been identified on the cell membranes of RPE cells, retinal glial cells, and fibroblasts (77). Activation of PDGFR drives the transformation of RPE cells, Müller cells, and fibroblasts into myofibroblasts (29, 76). Studies have detected PDGF and activated PDGFR in the retinal pigment epithelium, retinal glial cells, and vitreous of patients with PVR (78). Furthermore, PDGF-BB (an isoform of PDGF) stimulates proliferation and migration of cultured RPE cells by engaging the participation of mitogen-activated protein kinase and induces α-SMA expression (79).

Elevated VEGF expression is also observed in the vitreous of PVR patients. As PVR is an avascular disease lacking neovascularization, VEGF primarily promotes ECM deposition and fibrotic membrane formation by regulating the activity of RPE cells, fibroblasts, and transdifferentiated myofibroblasts (80, 81). VEGFA can competitively bind PDGFRα (indirect activation) with PDGF to participate in PVR (76). Indirectly activated PDGF receptors lead to reduced levels of tumor protein 53 (TP53). This reduction in TP53 promotes the survival of cells migrating into the vitreous and mediates the contraction of the PVR membrane formed by these cells (82). 

### 4.2 Expression of inflammatory cytokines and their related pathways

In the early stages of injury, TNF-α is upregulated as a key inflammatory cytokine, leading to immune cell recruitment and promoting the activation of retinal cells (83). Persistently elevated levels of TNF-α can trigger downstream pathways that promote the development of fibrosis (84). TNF-α primarily induces EMT in RPE cells by activating the NF-κB signaling pathway, thereby promoting the transcription of genes including snail, IL-6, and IL-8 (85). Additionally, TNF-α and TGF-β exhibit synergistic effects. Schiff et al. performed RNA sequencing on fibrous membranes resected from PVR patients, revealing activation of the p38-MAPK signaling pathway. Inhibition of p38 blocks TNF-α and TGF-β-mediated RPE cell transformation and membrane contraction, thereby reversing membrane contractility (86).

In addition to TNF-α, the release of several inflammatory cytokines such as IL-6, IL-8, IL-10, IL-1β, and IFN-γ significantly promotes the development of PVR (5, 87). 促The inflammatory environment serves as the critical first step in initiating the PVR disease process. Elevated levels of IL-6 and IL-8 have been reported in the vitreous of PVR patients (88, 89). Furthermore, in rabbit PVR models, a marked initial surge of inflammatory cytokines IL-6 and IL-8 in the vitreous was observed, with CRP and IFN-γ showing significant correlation with PVR severity. This indicates that inflammation not only initiates the PVR process but also exacerbates its severity (5). *In vitro*, IL-6 significantly induces RPE cell proliferation and EMT in a dose-dependent manner, accompanied by rapid phosphorylation of JAK1 and STAT3 (90). Furthermore, IL-6 enhances MMPs activity, contributing to ECM remodeling (91). As summarized in **Table 1**, a wide array of cytokines and growth factors, derived from diverse cellular sources, orchestrate the complex pathophysiology of PVR.

**Table 1:** Cytokines involved in PVR.

Molecular category	Molecular name	Primary cell sources	The primary role in PVR	References
Inflammatory factor	IL-1β	Macrophage	Promotes inflammatory responses, stimulates the expression of other inflammatory factors and MMPs, and participates in tissue destruction and remodeling.	(92, 93)
	IL-6	Macrophage, RPE cell, Müller cell	Induces EMT in RPE cells, promoting their proliferation and migration; enhances MMPs activity, contributing to ECM remodeling.	(90, 91)
	IL-8	RPE cell, glial cell, macrophage	Recruits inflammatory cells into the vitreous cavity and retina, chemotaxes fibroblasts, and stimulates fibroblast differentiation into myofibroblasts.	(94, 95)
	TNF-α	Macrophage, RPE cell	Enhance RPE cell migration capacity; Promote inflammatory cell infiltration; Induce the release of other cytokines.	(84, 85)
Growth factor	TGF-β	Macrophage, RPE cell, Müller cell	Strongly induces RPE cell EMT; significantly promotes ECM synthesis; inhibits ECM degradation; synergistically increases retinal traction with other factors.	(67, 70, 96)
	PDGF	Platelet, activated macrophage, RPE cell	Promotes migration and proliferation of RPE cells, Müller cells, and fibroblasts; synergistically enhances cell contraction and ECM production with TGF-β.	(30, 97)
	VEGF	RPE cell, Müller cell, macrophage, vascular endothelial cell	Promotes ECM deposition by modulating the activity of RPE cells, fibroblasts, and transdifferentiated myofibroblasts.	(80, 81)
	CTGF	RPE cell	Enhances the pro-fibrotic effects of TGF-β, promoting cell adhesion, proliferation, and ECM production.	(73, 74)
	bFGF	Multiple cell	Promote the proliferation and migration of various cell types, such as RPE cells and fibroblasts.	(98)
	HGF	Mesenchymal cell, macrophage	Promotes migration and proliferation of RPE cells; participates in inducing the EMT process	(99)
	EGF	Multiple cell	Promote the proliferation and migration of RPE cells	(100)

## 5 Other pathways promoting fibrosis

**Figure 3:**
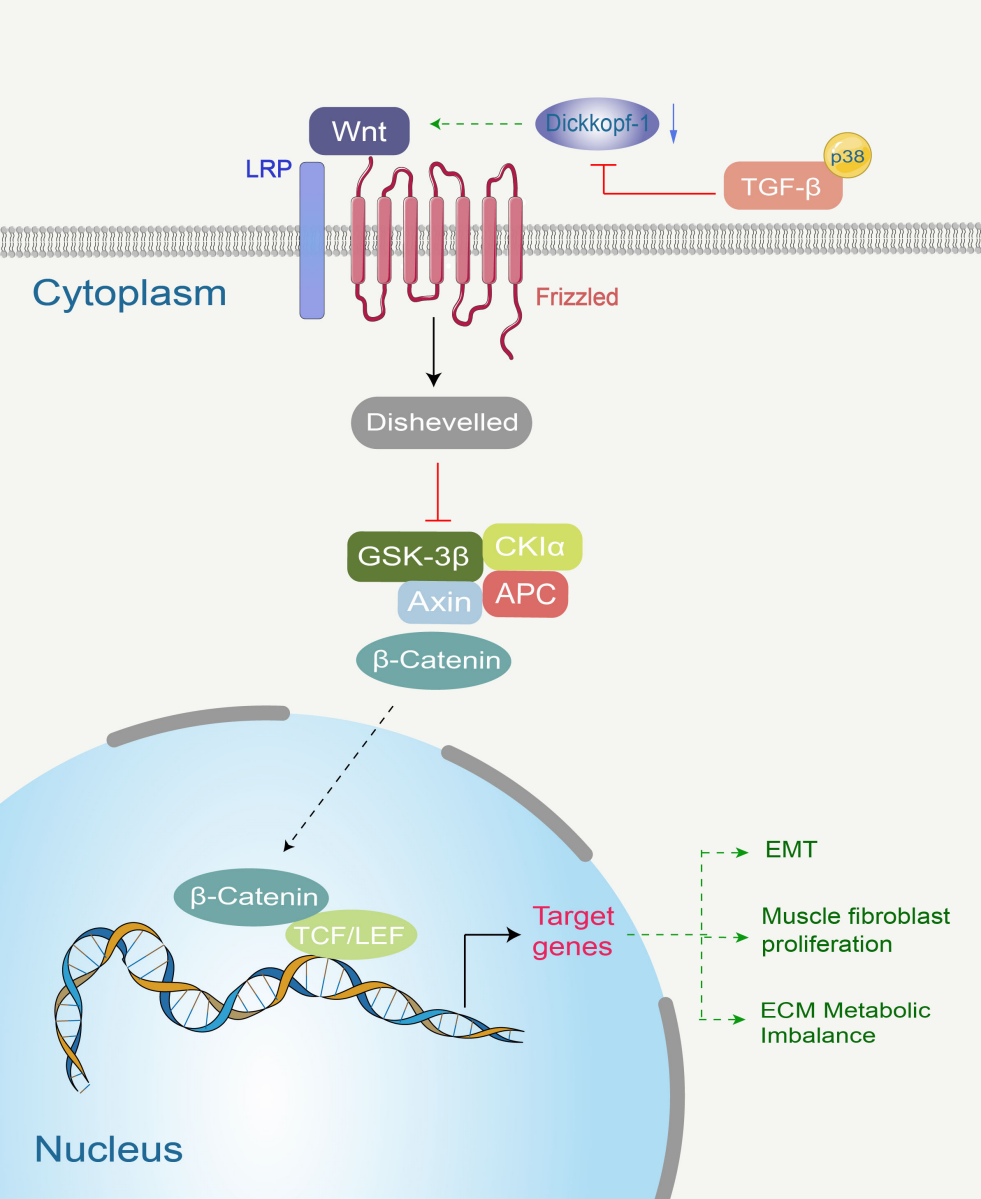
TGF-β stimulates the canonical Wnt signaling pathway in a p38-dependent manner by reducing the expression of the Wnt antagonist Dickkopf-1. Wnt proteins are secreted ligands that transmit their signals at the plasma membrane by interacting with their receptors Frizzled and the co-receptor LDL receptor-related protein (LRP5/6). Upon receptor binding, Wnt proteins induce a series of intracellular signaling events involving Disheveled, Axin, APC, CKI-α, and GSK-3β proteinsultimately leading to the stabilization of β-catenin. β-catenin translocates to the nucleus, where it binds to Transcription Factor/Lymphoid Enhancer Factor (Tcf/Lef) to induce transcription of Wnt target genes.

As mentioned earlier, TGF-β mediates EMT and fibrosis through both canonical and non-canonical pathways. However, inhibiting these downstream pathways does not completely eliminate TGF-β’s proliferative effects on fibroblasts, suggesting that additional pathways are crucial for transmitting TGF-β’s pro-fibrotic signaling. Research has revealed that TGF-β stimulates the canonical Wnt signaling pathway in a p38-dependent manner by downregulating the expression of the Wnt antagonist Dickkopf-1 (101, 102). Activation of the canonical Wnt pathway plays a significant role in the fibrotic process. Binding of Wnt-1 protein to cultured quiescent fibroblasts increases αSMA protein levels and induces stress fiber formation, indicating enhanced differentiation of quiescent fibroblasts toward myofibroblasts. Beyond Wnt-1, other Wnt proteins such as Wnt-3a also stimulate collagen release and induce differentiation of quiescent fibroblasts toward myofibroblasts (101). Notably, fibrosis induced by Wnt activation exhibits more pronounced effects than other fibrotic pathways. The classical Wnt pathway and its potent pro-fibrotic action suggest that Wnt signaling may serve as a potential target for novel anti-fibrotic strategies. **Figure 3** details the molecular cascade of the canonical Wnt pathway, highlighting how its activation by TGF-β leads to β-catenin stabilization and subsequent transcription of pro-fibrotic genes, underscoring its significance as a potential therapeutic target.

The PI3K/Akt signaling pathway is an intracellular signaling pathway that promotes metabolism, proliferation, cell survival, growth, and angiogenesis by responding to extracellular signals (103). Studies have confirmed that the vitreous in PVR patients can activate the PI3K/Akt signaling pathway in RPE cells *in vitro* (104). Research has identified PI3K expression in RPE cells, and PI3K inactivation prevents vitreous-induced proliferation, migration, and contraction of RPE cells (105).

The Notch signaling pathway is a crucial intercellular interaction pathway comprising Notch ligands, Notch receptors, and intracellular effector molecules, and is closely associated with organ fibrosis (106). Notch signaling regulates hypoxia-induced Müller cell proliferation and differentiation (107). The Notch signaling pathway plays a crucial role in TGF-β1-induced EMT *in vitro* and in mouse PVR models. Specific inhibition of Notch signaling via γ-secretase inhibitors may offer a novel approach for preventing PVR (108). Notably, Notch ligands and TGF-β1 exhibit synergistic effects on the overexpression of extracellular matrix proteins in Müller cells (109).

In recent years, novel pathways involved in PVR have been progressively identified, including the Nrf2 signaling pathway, RUNX1-related signaling pathways, and the RANK-NFATc1 signaling pathway. Studies indicate that Nrf2 was identified as a key sensor and effector in gene enrichment analysis of PVR animal models, and activating the Nrf2 signaling pathway can prevent retinal scar formation in experimental rabbit PVR (110). Furthermore, RUNX1 is highly expressed in proliferative membranes of PVR patients, and RUNX1 inhibitors suppress EMT in RPE cells (111, 112). Liao et al. further confirmed abnormal activation of the RANK-NFATc1 pathway following PVR. Targeted downregulation of NFATc1 in RPE cells effectively slowed disease progression (113). **Table 2** systematically catalogs the key signaling pathways implicated in PVR, detailing their core components and primary roles.

**Table 2:** Signaling pathways involved in the occurrence and development of PVR.

Signal pathway	Key molecules	The primary role in PVR	References
TGF-β signaling pathway	TGF-β1, TGF-β2, SMAD2/3	Induces RPE cell EMT, promotes fibrosis and ECM deposition; drives inflammation and fibrosis through classical and non-classical pathways.	(70, 71)
Wnt/β-catenin signaling pathway	Wnt-1, Wnt-3a, β-catenin, Frizzled receptor, LRP5/6, Dickkopf-1	Promotes fibrosis through TGF-β activation via a p38-dependent pathway; induces differentiation of quiescent fibroblasts into myofibroblasts.	(101, 102)
NOTCH signaling pathway	Notch ligand, Notch receptor, γ-secretase	Regulates hypoxia-induced Müller cell proliferation and differentiation; synergistically promotes ECM protein overexpression with TGF-β.	(106–109)
PI3K/AKT signaling pathway	PI3K, AKT, mTOR	Promotes proliferation, migration, and contraction of RPE cells; activated by vitreous fluid, participates in metabolism and cell survival.	(104, 105)
MAPK signaling pathway	p38, ERK, JNK	Mediates cellular stress responses and synergistically promotes EMT and membrane retraction with TGF-β and TNF-α.	(114, 115)
JAK/STAT signaling pathway	JAK1, STAT3	IL-6 induces RPE cell EMT and proliferation through this pathway.	(90)
Rho/ROCK signaling pathway	Rho, ROCK	Participates in the non-canonical TGF-β pathway, regulating the cytoskeleton and contraction.	(70, 71)
Nrf2 signaling pathway	Nrf2	As an oxidative stress sensor, it can prevent retinal scar formation upon activation.	(110)
RUNX1 signaling pathway	RUNX1	Highly expressed in PVR membranes, promoting EMT; inhibition of RUNX1 suppresses EMT in RPE cells.	(111, 112)
RANK-NFATc1 Signaling Pathway	RANK, NFATc1	Abnormally activated in traumatic PVR, promoting disease progression	(113)

## 6 Advances in pharmacotherapy for PVR

To provide a clear translational perspective, the following discussion on pharmacotherapeutic advances is structured according to the level of clinical evidence and intended clinical context. We first review interventions that have been evaluated in human clinical trials (primarily for prevention or adjunctive therapy), followed by promising agents currently supported by preclinical data (*in vitro* and animal models) that target specific pathogenic pathways of PVR.

### 6.1 General drug therapy

Currently, no drug interventions have been applied in the clinical treatment of PVR, likely due to concerns regarding efficacy and biosafety (116). Current drug therapies primarily target inflammatory, proliferative, and fibrotic pathways. For example, corticosteroid therapy targets broad inflammatory pathways; anti-VEGF therapies may exert anti-inflammatory effects by inhibiting immune cell chemotaxis (117); heparin modulates pro-fibrotic growth factors such as FGF and VEGF (118); and antimetabolites and related drugs like 5-FU and doxorubicin inhibit cell proliferation. However, a meta-analysis by Hunter et al. evaluated the efficacy and safety of PVR drug therapies following open-globe injury (OGI) and rhegmatogenous retinal detachment (RRD) across 27 randomized controlled trials (encompassing 3,375 patients), including interventions such as corticosteroids, antimetabolites, anti-VEGF agents, heparin, infliximab, methotrexate, and retinoic acid. Notably, these clinical trials have largely failed to demonstrate significant benefits in primary outcomes such as retinal reattachment rates or visual acuity improvement compared to standard surgical care alone.​ This highlights the challenge of translating broad-spectrum anti-inflammatory and anti-proliferative agents into clinical success for PVR (116).

A study investigated the efficacy of a 0.7 mg sustained-release dexamethasone vitreous implant as an adjunctive treatment for PVR (Grade C), finding fewer cases of cystoid macular edema at 6 months postoperatively. However, no difference was observed in anatomical or functional success compared to vitrectomy alone (119). Regarding PVR prevention, a recent randomized controlled trial showed no benefit—and even a negative impact on surgical outcomes—from adding triamcinolone acetate during vitrectomy in patients with open-eye trauma (120). Additionally, Rajan et al. employed repeated intravitreal methotrexate injections to prevent postoperative RRD recurrence in PVR patients, yet observed no difference in overall re-detachment rates or BCVA (121). Another study employed PVR adjunctive therapy with 5-FU combined with low molecular weight heparin (LMWH), but found no improvement in either the anatomical or visual outcomes of macular-involving retinal detachments. Conversely, patients with macula-sparing retinal detachments experienced poorer visual outcomes (122).

To provide deeper insight into these neutral or negative outcomes, a structured analysis of potential contributing factors is crucial. Several key variables in trial design may have significantly influenced the results. Firstly, patient selection and disease stage are critical; trials often enroll patients with varying degrees of PVR severity (e.g., Grade C vs. established PVR), yet the pathobiology and therapeutic susceptibility likely differ between early inflammatory/proliferative phases and late fibrotic stages. Secondly, the timing of intervention is paramount. Administering anti-proliferative or anti-fibrotic agents after fibrous membrane formation may be too late to reverse established traction. Lastly, the choice of clinical endpoints (e.g., anatomical success vs. functional visual improvement) may not fully capture the biological effect of a drug if the primary driver of final visual outcome remains surgical complexity. A more nuanced consideration of these factors is essential for designing future trials.

Although these anti-inflammatory and anti-fibrotic drugs appear to show promising results in preclinical models in some other studies (123, 124), they remain highly controversial and have not been widely adopted as adjunctive therapies for PVR in clinical practice. Given the varying severity and progression stages of PVR among patients, the efficacy of these drugs is likely to differ significantly between individuals. Second, these drugs lack specificity, exhibit short intraocular half-lives, and may induce side effects when acting on other ocular structures (17, 18). Therefore, developing novel drugs or targeted delivery systems based on the pathophysiology of PVR represents a current and future research priority.

### 6.2 Pathway inhibitors targeting the mechanism of PVR

Unlike the aforementioned clinical trial drugs, the following candidate drugs represent highly promising preclinical strategies capable of precisely targeting key pathways in the pathogenesis of PVR, such as the TGF-β, MAPK, and PI3K/AKT pathways. It should be emphasized that the efficacy and safety data for these drugs currently derive primarily from *in vitro* and animal studies, and their translational potential remains to be validated in human trials.

Research has found that doxycycline can inhibit p38/MAPK activation and total MMP activity in RPE cells, thereby reducing their proliferation, migration, adhesion, and contraction (114). Another study found that neferine (a bis-benzylisoquinoline alkaloid) inhibits the progression of PVR by suppressing proliferation and migration of RPE cells through downregulating p38/MAPK and PI3K/AKT signaling pathways (115). Wang et al. found that demethoxycurcumin (a curcuminoid) inhibits RPE cells migration and MMP-2 expression by downregulating the STAT-3 signaling pathway (125). Zhang et al. demonstrated that crocetin (an antioxidant carotenoid abundant in saffron) inhibits PDGF-BB-induced activation of PDGFRβ and its downstream PI3K/Akt, ERK, p38, and JNK pathways, effectively suppressing PDGF-BB-induced RPE cell proliferation and migration (126). Our previous studies confirmed that artesunate inhibits TGF-β/Smad signaling and PI3K/AKT pathways in PVR to suppress RPE cells proliferation and migration (127, 128). Subramani et al. found that bevacizumab reduces Notch signaling pathway expression, thereby affecting RPE cells function (129). Furthermore, a heavy-chain hyaluronic acid/succinic acid 3 purified from human amniotic membrane inhibits RPE cell proliferation and EMT by downregulating Wnt/β-catenin and TGF-β/Smad signaling pathways (130).

These drugs can precisely target one or more pathways involved in the PVR development process, thereby inhibiting its progression. However, translating these drugs into clinical applications remains challenging. Current research is still confined to cellular and animal studies, and future translational research must prioritize evaluating drug safety, pharmacokinetic properties, and ultimate therapeutic efficacy in humans.

### 6.3 The potential of drug delivery systems in PVR treatment

**Figure 4:**
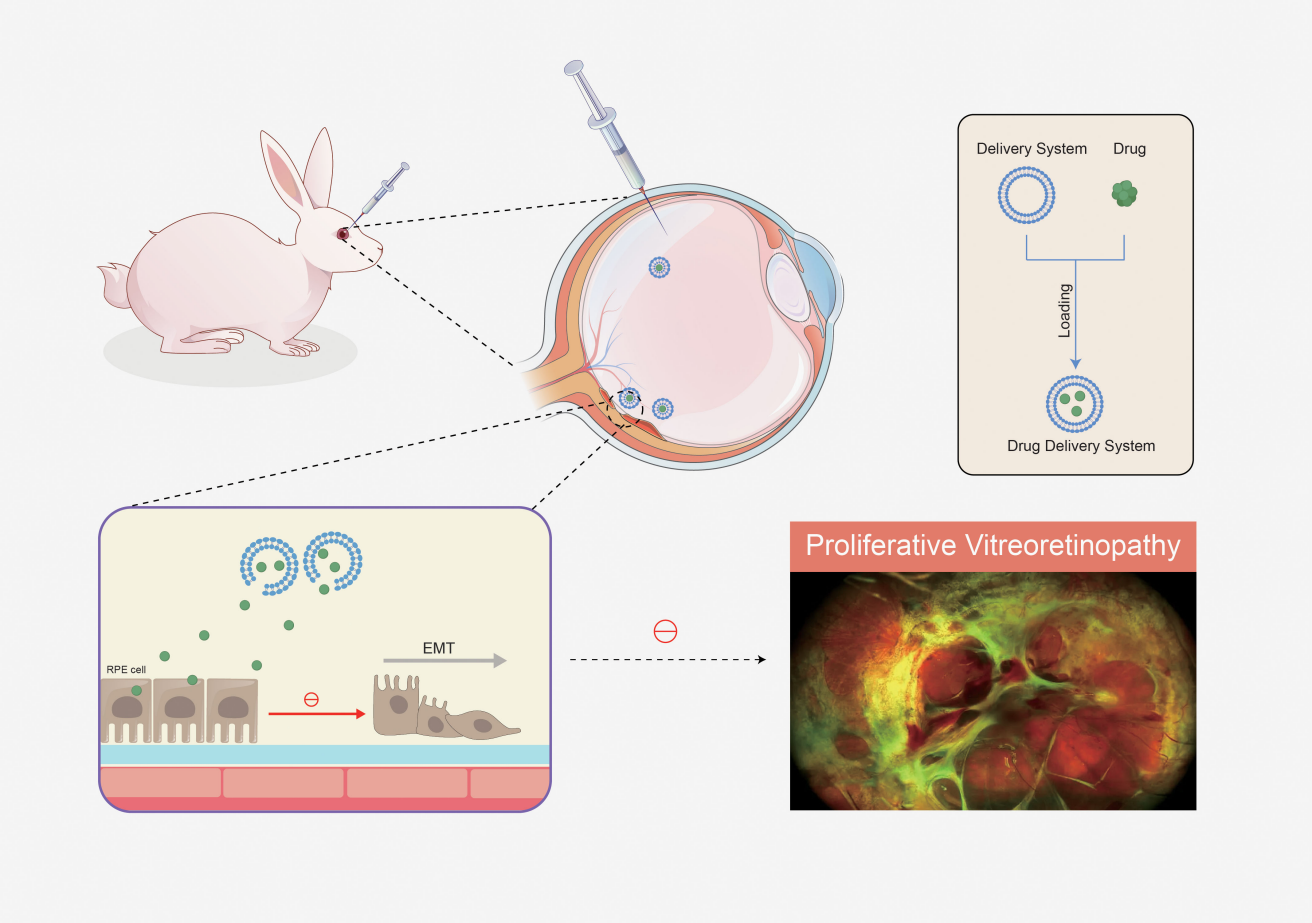
Mechanism of action of drug delivery systems.

For years, extensive research has focused on developing drug delivery systems to enhance drug bioavailability at target sites while minimizing side effects. Researchers have investigated and developed innovative drug carrier systems designed to precisely target specific locations, prolong drug retention time, reduce administration frequency, improve therapeutic efficacy, and ensure biocompatibility. These advancements effectively address the current limitations of drug therapy for PVR (131). Although still in the preclinical development stage, the following platforms offer innovative solutions for sustained targeted therapy. The mechanisms by which advanced drug delivery systems aim to overcome pharmacological limitations are depicted in **Figure 4**.

Current research primarily focuses on utilizing nanomaterials as drug delivery vehicles for PVR treatment. Li et al. employed polyethylene glycol-block-polyϵ-caprolactone (PEG-b-PCL) micelles for *in vitro* delivery of dasatinib to treat PVR. They demonstrated that PEG-b-PCL microspheres loaded with dasatinib inhibited proliferation, migration, and adhesion of RPE cells, exhibiting good biocompatibility and no ocular toxicity (132). Wu et al. developed an engineered episcleral drug film using biodegradable material, Poly(L-lactide)-co-poly(ε-caprolactone), and triamcinolone acetonide (TA) as a model drug. This film can be easily applied over a ruptured sclera during initial trauma repair surgery, enabling sustained release of therapeutic doses for up to six months without toxicity. In the rabbit eye PVR model, the drug-coated film group significantly reduced the risk of severe proliferative retinopathy by 5.7-fold compared to the drug-free film control group (133). Xiao et al. employed a controlled-release system using porous silica to co-deliver daunorubicin and dexamethasone, demonstrating good tolerability following intraocular injection in rabbits. Compared to carriers delivering either daunorubicin or dexamethasone alone, this controlled-release system exhibited significantly superior performance in inhibiting cell proliferation and PVR-associated markers. Furthermore, the formulation sustained drug release within the eye for at least 90 days (134).

Several specialized drug delivery systems have been reported. Several specialized drug delivery systems have been reported. Yu et al. developed a hydrogel using crosslinked polyvinyl alcohol (PVA) and chitosan as a potential vitreous substitute. They encapsulated 5-FU within a poly(lactic-co-glycolic acid) (PLGA) copolymer and loaded it onto the PVA/chitosan gel for treating PVR. Animal studies demonstrated that this vitreous substitute effectively suppressed RPE cell proliferation, significantly reduced recurrence rates, and maintained physical integrity after 24 weeks (135). Chen et al. prepared a lipid prodrug of PMEG (a potent inhibitor of DNA synthesis)—hexadecyloxypropyl9-[2-(phosphonomethoxy)ethyl]guanine(HDP-PMEG) —to evaluate its efficacy as a pharmacological adjunct for PVR surgical treatment. Following intravitreal injection of 3 µg into rabbit eyes, HDP-PMEG significantly inhibited laser-induced fibro-vascular proliferation in rat eyes by up to 55%. The drug persisted in rabbit vitreous for at least 6 weeks, demonstrating a marked inhibitory effect on PVR formation (136). Wang et al. developed a novel approach using retinoic acid-loaded sodium alginate microspheres for treating retinal diseases. Its stability, sustained-release properties, and biocompatibility suggest suitability for long-term therapy, demonstrating significant potential in PVR treatment (137).

Exosomes, a hot research topic in recent years, hold great potential in PVR drug delivery systems. ZExosomes, a hot research topic in recent years, hold great potential in PVR drug delivery systems. Zhao et al. co-loaded doxorubicin and dexamethasone into RPE cell-derived exosomes, creating an Exos-based dual-drug nanocarrier (Exos@D-D) for targeted PVR therapy. They found that Exos@D-D exhibited strong RPE targeting and enhanced uptake efficiency, effectively inhibiting RPE proliferation, migration, and EMT. In animal studies, it also significantly suppressed proliferative membrane formation, demonstrating remarkable therapeutic efficacy against PVR while maintaining excellent biocompatibility (7).

In summary, the advanced drug delivery systems discussed herein, including nanomaterials, hydrogels, and exosomes, represent a paradigm shift in PVR pharmacotherapy. By enabling targeted, sustained, and controlled release of therapeutic agents, these platforms hold the promise to overcome the fundamental limitations of conventional intravitreal injections, such as rapid clearance and poor bioavailability. While most of these systems are still in the preclinical stage, their continued development and optimization are crucial for translating potent pathway inhibitors from bench to bedside, ultimately paving the way for more effective and safer adjunctive therapies for PVR.

## 7 Conclusion

Overall, surgery remains the primary treatment for PVR, but its efficacy is limited and carries a risk of recurrence. Currently, no drugs have been formally approved for the prevention or treatment of clinical PVR. Non-surgical interventions studied thus far—such as corticosteroids and antimetabolites—have failed to significantly improve outcomes in clinical trials. Future research should focus on developing innovative, multi-targeted therapies with precise drug delivery mechanisms for PVR, while prioritizing both safety and efficacy, to achieve more effective prevention and treatment of PVR.
